# Hyperglycemia-induced accumulation of advanced glycosylation end products in fibroblast-like synoviocytes promotes knee osteoarthritis

**DOI:** 10.1038/s12276-021-00697-6

**Published:** 2021-11-10

**Authors:** Qingxian Li, Yinxian Wen, Linlong Wang, Biao Chen, Jun Chen, Hui Wang, Liaobin Chen

**Affiliations:** 1 Division of Joint Surgery and Sports Medicine, Department of Orthopedic Surgery, Zhongnan Hospital of Wuhan University, Wuhan, 430071 China; 2 Hubei Provincial Key Laboratory of Developmentally Originated Disease, Wuhan, 430071 China; 3 Joint Disease Research Center of Wuhan University, Wuhan, 430071 China; 4 Department of Pharmacology, School of Basic Medical Sciences, Wuhan University, Wuhan, 430071 China

**Keywords:** Glycobiology, Mechanisms of disease

## Abstract

Osteoarthritis (OA) is significantly associated with diabetes, but how hyperglycemia induces or aggravates OA has not been shown. The synovium plays a critical role in cartilage metabolism and substance exchange. Herein, we intended to investigate whether and how hyperglycemia affects the occurrence and progression of OA by influencing the synovium. In patients with knee OA and diabetes (DM OA), we found a more severe inflammatory response, higher endoplasmic reticulum stress (ERS) levels, and more advanced glycosylation end products (AGEs) accumulation in the synovium than in patients without diabetes. Subsequently, we found similar results in the DM OA group in a rat model. In the in vitro cocultivation system, high glucose-stimulated AGEs accumulation, ERS, and inflammation in rat fibroblast-like synoviocytes (FLSs), which resulted in chondrocyte degeneration due to inflammatory factors from FLSs. Furthermore, in the synovium of the DM OA group and FLSs treated with high glucose, the expression of glucose transporter 1 (GLUT1) and its regulatory factor hypoxia-inducible factor (HIF)-1α was increased significantly. Inhibitors of HIF-1α, GLUT1 or AGEs receptors attenuated the effect of high glucose on chondrocyte degradation in the FLS-chondrocyte coculture system. In summary, we demonstrated that hyperglycemia caused AGEs accumulation in FLSs via the HIF-1α-GLUT1 pathway, which increases the release of inflammatory factors from FLSs, subsequently inducing chondrocyte degradation and promoting OA progression.

## Osteoarthritis: clarifying the links to diabetes

High blood sugar caused by diabetes can promote the progression of osteoarthritis (OA) by triggering joint-damaging inflammation. Although OA is known to be linked to diabetes, the mechanism was unclear. Liaobin Chen and Hui Wang at Wuhan University in China and coworkers investigated how high blood sugar affects OA progression in the synovium, the membrane surrounding the fluid-filled capsule inside the knee and other joints. The researchers found that the synovium was more inflamed in patients with diabetes and OA than in patients with OA alone. Investigation in a rat model showed that high blood sugar levels caused accumulation of sugar–protein complexes (AGEs) that trigger inflammation, and of a protein that transports sugar (GLUT1). Blocking GLUT1 in cultured cells decreased AGEs accumulation, flagging it as a potential target for developing treatments for knee OA.

## Introduction

Osteoarthritis (OA) is the major cause of joint pain in the elderly, and the prevalence was reported to be 15–18% in the whole population^1^ and even as high as 50% in those over 60^2^. Data from the International Diabetes Federation showed that approximately 463 million diabetes mellitus (DM) patients had been diagnosed in 2017, with the number predicted to reach 700 million in 2045^3^. Thus, both OA and DM have become major public health concerns. Moreover, DM, one of the major metabolic syndromes (MetS), was identified as an independent risk factor for knee OA by epidemiological studies and could even trigger and aggravate the progression of knee OA^4,5^. Animal experiments also demonstrated an insulin-like growth factor 1-resistant state in the cartilage of rats with DM induced by streptozotocin (STZ)^6^. Such findings indicated a tight correlation between OA and DM. However, whether and how hyperglycemia aggravates or even triggers OA pathogenesis is still unclear.

Synovitis is often the initial pathological change of OA and may occur before visible changes in the cartilage^7^. As a major population of synovial cells, fibroblast-like synoviocytes (FLSs) are believed to participate in OA progression by secreting inflammatory factors, including interleukins (ILs) and tumor necrosis factors (TNFs), as well as matrix metalloproteinases (MMPs) and ADAM metallopeptidase with thrombospondin type (ADAMTS) proteases, which modulate the components of cartilage extracellular matrix (ECM)^8^. Studies have indicated that hyperglycemia induces inflammation in multiple tissues, including the kidney, retina, and blood vessels^9,10^. Additionally, collagen I accumulation was observed in the synovial tissue of rats with STZ-induced DM^11^, while catabolic and inflammatory responses were observed in primary mouse chondrocytes treated with high glucose^12,13^. As the synovium has a better blood supply than the cartilage and is much more sensitive to serum regulators, we believe that the synovium, rather than the cartilage, might be the major target tissue of hyperglycemia during the pathogenesis of DM-related OA^14^.

As one of the most essential glucose transporters (GLUTs) located on the surface of the cell membrane, GLUT1 determines the energy metabolism of mammalian cells^15^. GLUT1 was found to act in concert with cytokines and growth factors to induce diabetic glomerulosclerosis^16^, while the expression and function of GLUT1 were also stimulated in human FLSs with rheumatoid arthritis, as well as a mouse model of inflammatory arthritis^17,18^. The stimulated function of GLUT1 might further induce the accumulation of advanced glycosylation end-products (AGEs)^19^ and subsequently induce endoplasmic reticulum stress (ERS) and the release of inflammatory factors^20,21^. However, whether GLUT1 participates in DM-related OA is still unknown.

To clarify the relationship between hyperglycemia and OA, as well as the role of FLSs in the progression of OA, we collected human synovium from OA patients with or without DM, generated a rat model of DM-related OA, and prepared high glucose-stimulated rat FLS cultures in vitro to explore the role of FLSs in DM-related OA and the mechanism, involving GLUT1 and AGEs.

## Materials and methods

### Chemicals and reagents

This information is shown in the supplementary materials.

### Human synovial specimens

To observe the effect of DM on the synovium, we obtained specimens of the knee synovium from individual OA patients with or without DM during total knee arthroplasty (TKA) surgery from January 1st, 2019 to January 1st, 2021 (*n* = 10 samples/group, aged between 50 and 70 years old, grade 4 by Kellgren & Lawrence classification for knee OA). The basic patient information is presented in Supplementary Table S1 and Supplementary Table S2. The exclusion criterium for the specimens was a history of knee surgery or a history of intra-articular drug injection to ensure that the original synovial tissue was harvested. The collected specimen was transferred to a 0.9% NaCl solution and then transferred to a 4% paraformaldehyde solution after trimming for follow-up morphological examination. The protocol used in this study complies with the ethical guidelines of the Helsinki Declaration in 1975 and was approved by the Medical Ethics Committee Zhongnan Hospital, Wuhan University (No. 2019018). The patient’s written informed consent was obtained for all operations involved.

### Rat DM model and OA model establishment

Forty specific pathogen-free Wistar rats (No. 2010–2012, certification number: 42000600014526, license number: SCXK) (Hubei, China) with males weighing 280 ± 20 g were obtained from the Experimental Center of the Hubei Medical Scientific Academy (Wuhan, China). All of the animal experimental procedures were conducted according to the Guidelines for the Care and Use of Laboratory Animals of the Chinese Animal Welfare Committee. This study was approved by the Animal Experimental Ethics Committee of Wuhan University Medical College (license number: 14016). The animals were housed in padded cages in an air-conditioned room under standard conditions (room temperature: 18–22 °C; humidity: 40–60%; light cycle: 12 h light-dark cycle; and 10–15 air changes per hour) and allowed free access to rat chow and tap water. All rats were acclimated one week before experimentation.

After one week of adaptive feeding, 40 rats were randomly divided into a DM group (*n* = 20) and a control group (*n* = 20). Rats in the DM group were intraperitoneally injected with 60 mg/kg STZ^22^. Normal saline was injected intraperitoneally into the control rats (0.3 ml/100 g). Two weeks later, blood glucose was measured through the tail puncture, and rats with fasting blood glucose greater than 13.3 mM were considered successful DM model rats^23^. One month after DM modeling, half of the rats in the DM and control groups were randomly selected to experience long-distance treadmill running; the other half continued the usual feeding. A brief description of the animal treatment is shown in Fig. 2a.

The long-distance treadmill running experiment was divided into six cycles, with each cycle 7 days in duration^24^. The total accumulated mileage was 30 kilometers. After running, the rats were sacrificed, and joint samples in both knees were harvested. Articular cartilage from one knee was ink-stained to observe morphological changes, and cartilage from the other joint was fixed in neutral formalin. After decalcification, the joint specimens were embedded in paraffin and sliced for safranin O staining to observe pathological changes, which were subsequently evaluated with Mankin’s scoring.

### Pathological scoring of OA and synovitis

According to the modified Mankin’s score standard^25^ (the grading system was composed of four categories—cartilage structure (6 points), cartilage cells (3 points), staining (4 points) and tidemark integrity (2 points)—with the highest score of 14 points; normal cartilage scored 0), safranin O-stained slices of knee joints from the rats with or without running were all scored. The severity of OA in the experimental rats was evaluated quantitatively from the aspects of cartilage structure, cell number, matrix staining, and tidal line.

According to the synovial pathology score standard^26^ (0–1, no synovitis; 2–4, low-grade synovitis; 5–9, high-grade synovitis), synovial H&E slices from human synovial specimens and rats with or without running were all scored. From the aspects of the synovial lining cell layer, intrinsic cell density, and inflammatory infiltration, the severity of knee synovitis was evaluated quantitatively.

### Transwell coculture experiment of rat primary chondrocytes and FLSs

The experimental animals were sacrificed after successful isoflurane anesthesia, and the synovial tissue and cartilage of the knee joint were separated with an aseptic operation. Synovial tissue and cartilage were fully chopped under sterile conditions and then digested with 0.2% type II collagenase solution at 37 °C in a 5% CO_2_ incubator for 6–8 h, centrifuged at 120 × g for 5 min and resuspended in DMEM containing 10% FBS. Primary cells were seeded at a density of 1.0 × 10^5^ cells/ml. The cells were cultured with DMEM (with 10 mM glucose) containing 10% FBS. Then, 100 U/ml penicillin and 50 μg/ml streptomycin were added to the above culture system. Subsequent experiments were performed with third- to fourth-generation primary cells.

To simulate the physiological relationship between the synovium and cartilage and investigate the influence of soluble factors released by FLSs on chondrocytes, we established a no-contact coculture model. Transwell cell culture inserts (no: 3450, pore size: 0.4 μm; Corning Costar Corp., NY, USA) were used in this study. FBS (10%) and antibiotics (1%) were placed in DMEM in the upper and lower compartments. The heights of the medium in the upper and lower compartments were maintained at similar levels. To investigate and highlight the role of the synovium in the development of DM-related knee OA in the current study, we resuspended chondrocytes at 5 × 10^4^ cells/well in the lower chamber (a 6-well plate) in control conditions (10 mM) to minimize the effect of high glucose on chondrocytes, and cell-attached slides were placed in the lower well if the chondrocytes were used for cellular immunofluorescence staining (IF); FLSs were seeded at 5 × 10^4^ cells/well in the upper chambers. FLSs were treated with different concentrations of glucose (10, 20, 30, and 40 mM) or other treatment factors. There was communication between different cells in the system through a polyester (PET) membrane for 72 h. The medium was refreshed every 24 h. All cell culture experiments were performed at 37 °C under normoxic and 5% CO_2_ conditions. The coculture experimental groups included coculture of chondrocytes with 10 mM glucose and FLSs with 10, 20, 30 and 40 mM glucose, coculture of chondrocytes with 10 mM glucose and FLSs with 10 mM glucose, 30 mM glucose, 10 mM glucose plus 2.5 μM 2-MeOE2, 30 mM glucose plus 2.5 μM 2-MeOE2, 10 mM glucose plus 0.5 μM STF-31, and 30 mM glucose plus 0.5 μM STF-31. All cocultures were set up in triplicate. For in vitro experiments, similar results were obtained in 6 independent experiments, and there were 3 repeats within an experiment.

### Preparation of pharmaceutical solvents

For different concentrations of glucose, we weighed the anhydrous glucose powder and dissolved it in 5 ml of glucose-free DMEM and filtered and sterilized and mixed it into the remaining medium. Then, we added 10% FBS, 100 U/ml penicillin, and 50 μg/ml streptomycin and finally generated a complete medium with 10, 20, 30, and 40 mM glucose.

AGE-BSA and its control BSA were dissolved in water and added to a complete medium to prepare a working solution with a final concentration of 100 ng/ml. Ti was dissolved in water and added to a complete medium to prepare a working solution with final concentrations of 0, 1, 3, and 5 μg/ml. We dissolved 4-PBA in water and added it to the complete medium to prepare a working solution with final concentrations of 0, 50, 100, 250, and 500 μM. We dissolved 2-MeOE2 in DMSO and added it to the complete medium to prepare a working solution with final concentrations of 0, 0.1, 0.25, 0.5, 1, 2.5, 5, 10, and 20 μM (the final concentration of DMSO was 0.1%, and the same volume of control medium containing 0.1% DMSO was added to the control cells). STF-31 was dissolved in water and added to complete medium to prepare a working solution with final concentrations of 0, 0.25, 0.5, 1, 2, 4, 6, 8, and 10 μM (the final concentration of DMSO was 0.1%, and the same volume of control medium containing 0.1% DMSO was added to the control cells).

### CCK-8 experiment

According to the manufacturer’s instructions, 5000 cells/100 ml was added to each well of a 96-well plate. After 24 h of cell attachment, 100 μl of complete medium containing different concentrations of 2-MeOE2, STF-31, Ti, and 4-PBA was added for cytotoxicity experiments. The samples were placed in a 37 °C and 5% CO_2_ incubator for cultivation. After 24, 48, or 72 h, 10 μl of enhanced CCK-8 solution was added to each well. Three blank controls (100 μl drug-containing medium and enhanced CCK-8 solution, without cells) were used for each experimental group. After incubation for 2.5 h in the cell incubator, the absorbance at 450 nm wavelength was measured with a microplate reader, and appropriate time points and drug concentrations were selected for subsequent experiments. After the cytotoxicity experiment, we determined that the appropriate administration time of 2-MeOE2 was 48 h and the appropriate concentration was 2.5 μM; for STF-31, the concentration was 0.5 μM and the time was 72 h; for 4-PBA, the concentration was 250 μM and the time was 48 h, and for Ti, the concentration was 1 μg/ml and the time was 48 h.

### Total RNA extraction and real-time qPCR (RT-PCR)

Total RNA was isolated from chondrocytes and FLSs using TRIzol reagent following the manufacturer’s protocol. The isolated RNA was stored at −80 °C in aliquots. An Applied Biosystems TaqMan Reverse Transcription kit was used to convert mRNA to cDNA. RT-qPCR was then performed using a SYBR Green qPCR Master Mix Kit and an ABI Step One Plus cycler (Applied Biosystems, Foster City, CA, USA) at 95 °C for 40 cycles of 15 s and 60 °C for 30 s. The expression of *Mmp3, Mmp13, Adamts4, Adamts5, Tnf, Il-6*, and *Slc2a1* relative to the expression of *β-actin* was calculated by the 2^-ΔΔCt^ method for standardization. Real-time PCR primers were designed with Primer Premier 6.0 (PREMIER Biosoft International, Palo Alto, CA, USA). Each of the designed primer sequences was queried using the NCBI BLAST database for homology comparisons to determine the final primers used in this study. All primers were synthesized by Sangon Biotech Co., Ltd. (Shanghai, China). The primer sequences and annealing temperatures for each gene are listed in Table 1.

**Table 1:** Oligonucleotide primers and PCR conditions in real-time quantitative PCR.

Gene	Forward primer	Reverse primer
*β-actin*	GGACCTGACAGACTACCTCA	GTTGCCAATAGTGATGACCT
*Mmp3*	TGGGAAGCCAGTGGAAATG	CCATGCAATGGGTAGGATGAG
*Mmp13*	TGACCTGGGATTTCCAAAAGAG	GTCTTCCCCGTGTCCTCAAA
*Adamts4*	TCGCTTCGCTGAGTAGATTCGT	TTCGGATGCTTGGATGCTTAA
*Adamts5*	CTGCGCTGTGATTGAAGATGAT	TGCTGGTAAGGATTGAAGACATT
*Tnf-α*	GCCACCACGCTCTTCTGT	GGCAGCCTTGTCCCTTGA
*Il-6*	CACTGCCTTCCCTACTTC	GCATCATCGCTGTTCATAC
*Slc2a1*	TCGTCGTTGGGATCCTTATTG	GAAGATGACACTGAGCAGTAGAG
*Col2a1*	GAGGGCAACAGCAGGTTCAC	GCCCTATGTCCACACCAAATTC
*Acan*	TGGCATTGAGGACAGCGAAG	TCCAGTGTGTAGCGTGTGGAAATAG

*β-actin* beta actin, *Slc2a1* solute carrier family 2 member 1, *Col2a1* α1 chain of type II collagen gene, *Acan* aggrecan, *Mmp3* matrix metallopeptidase 3, *Mmp13* matrix metallopeptidase 13, *Adamts4* ADAM metallopeptidase with thrombospondin type 1 motif-4, *Adamts5* ADAM metallopeptidase with thrombospondin type 1 motif-5, *Tnf-α* tumor necrosis factor, *Il-6* interleukin-6.

### H&E staining

Synovial specimens from humans and the complete articular tissue of rats were fixed in 4% paraformaldehyde (PFA) solution for 48–72 h and embedded in paraffin for subsequent H&E staining. Specifically, the tissue was fixed on paraffin sections, dehydrated, and embedded in paraffin. Then, the paraffin-embedded samples were cut into 5 μm serial sections, stained with hematoxylin dye for 5 min and washed with water. Next, the sections were soaked with ammonia and then washed with water. The sections were stained with 1% eosin dye and then washed with water. Ten different fields of view were chosen for each sample, and all images were captured on a Nikon NIS Elements BR light microscope (Nikon, Tokyo, Japan).

### Safranin O staining

For the rat knee joint, paraffin sections were deparaffinized in water. The sections were sequentially placed in xylene I-xylene II-anhydrous ethanol I-anhydrous ethanol II-75% alcohol and then washed with tap water. The slices were stained with safranin O for 15–30 s and quickly dehydrated with absolute ethanol. Clear xylene was applied to transparent sections for 5 min, and they were sealed with neutral gum.

Chondrocytes were washed with PBS 3 times after discarding the medium, 1 ml of safranin O dye was added to each well, and the cells were stained for 30–60 s, quickly dehydrated with absolute ethanol, and then observed and photographed as soon as possible. All of the images were captured and then analyzed using a Nikon NIS Elements BR light microscope (Nikon, Tokyo, Japan). The staining intensity was determined by measuring the integrated optical density (IOD) in 10 different fields for each sample.

### Immunohistochemical (IHC) staining

Synovial specimens of humans and complete articular tissues of rats were fixed in 4% paraformaldehyde solution for 48 h and treated with a paraffin-embedding technique. The paraffin samples were sectioned at 5 μm for morphological staining analysis. After dewaxing, rehydration, and antigen retrieval, paraffin sections were treated using EDTA antigen-repairing buffer (pH 8.0). BSA was used to block the previously added primary antibody, and the primary antibody dilution ratio was anti-MMP13 (1:50 dilution), anti-ADAMTS5 (1:50 dilution), anti-GRP78 (1:50 dilution), anti-ATF6 (1:50 dilution), anti-TNF-α (1:50 dilution), anti-IL-6 (1:50 dilution), anti-NF-κB p65 (1:50 dilution), anti-GLUT1 (1:250 dilution), anti-AGEs (1:250 dilution), and anti-HIF-1α (1:250 dilution). Immunohistochemistry was performed with a DAB staining kit (GeneTech Company, Ltd., Shanghai, China). All of the images were captured using a Nikon NIS Elements BR light microscope (Nikon, Tokyo, Japan). ImageJ 6.0 software was used to analyze the immunohistochemistry results. The staining intensity was determined by measuring the IOD in 10 different fields for each sample.

### Cellular IF staining

After 72 h of treatment, the cells cultivated in confocal dishes (or cell-attached slides) were washed three times with ice-cold PBS, fixed in 4% formaldehyde, and blocked for 30 min with 3% BSA in 0.2% Triton X-100/PBS. The cells were then incubated overnight at 4 °C with primary antibodies in blocking buffer, including rabbit anti-α1 chain of type II collagen gene (COL2A1) (1:200 dilution), anti-aggrecan (ACAN) (1:200 dilution), anti-AGEs (1:250 dilution), and anti-GLUT1 (1:50 dilution). The cells were washed with PBS, and a fluorescent secondary antibody Cy3-conjugated goat antirabbit IgG (H + L) (1:100 dilution) was added for 2 h at room temperature. The dishes or slides were washed four times with PBS, and the cells were then incubated with DAPI for 5 min at room temperature. The cells were washed twice with PBS, and fluorescence images were captured using a confocal microscope (Leica-LCS-SP8-STED, Leica, Germany). The staining intensity was determined by measuring the IOD in 10 different fields for each sample.

### Western blot

Briefly, cells were rinsed with ice-cold PBS and then lysed for 30 min at 4 °C in RIPA lysis buffer containing phosphatase inhibitor cocktail, followed by analysis with the BCA Assay Kit for protein quantification. A total of 30 μg of protein was loaded into each lane, isolated by SDS-PAGE (10% gels), and blotted onto PVDF membranes (Millipore, MA, USA). The membranes were blocked in 5% nonfat milk for 1 h and incubated overnight at 4 °C with the primary antibody. The dilution concentrations of the primary antibodies were anti-GRP78 (1:1000) and anti-ATF6 (1:1000). Then, the membranes were incubated with horseradish peroxidase (HRP)-conjugated secondary antibody (goat antirabbit IgG, 1:5000 and goat anti-mouse IgG, 1:5000) for 1 h and visualized using ECL HRP substrate (PerkinElmer, Inc., Boston, MA, USA). The antibody binding signals were detected using a ChemiDoc Image Analyzer (Bio-Rad, Hercules, CA, USA). The relative protein level was standardized with the ACTB (anti-ACTB, 1:100000) protein level. The protein band intensities were analyzed by ImageJ software from three independent bands.

### Statistical analysis

Prism (GraphPad Software, La Jolla, CA, USA, version 8.0) was used for all data analyses. All data values shown are presented as the means±S.E.M. For in vitro experiments, data with different drug concentrations were analyzed using one-way analysis of variance (ANOVA) with the post hoc test for multiple comparisons. Unpaired Student’s *t*-test was applied for comparisons between two groups if the homogeneity of variance was consistent. Satterthwaite’s *t* test was used if the homogeneity of variance was not equal. In the human and animal experiments, a sample size calculation in each group was based on a power analysis with *β* = 0.8 and *α* = 0.05 to detect a significant difference among groups. For the human and animal experiments, we used the Mann–Whitney *U* test for comparisons between the non-DM OA (control) and DM OA (DM) groups. *P* < 0.05 was considered statistically significant for all tests.

## Results

### More severe inflammatory response of the articular synovial membrane in OA with DM

**Fig. 1:**
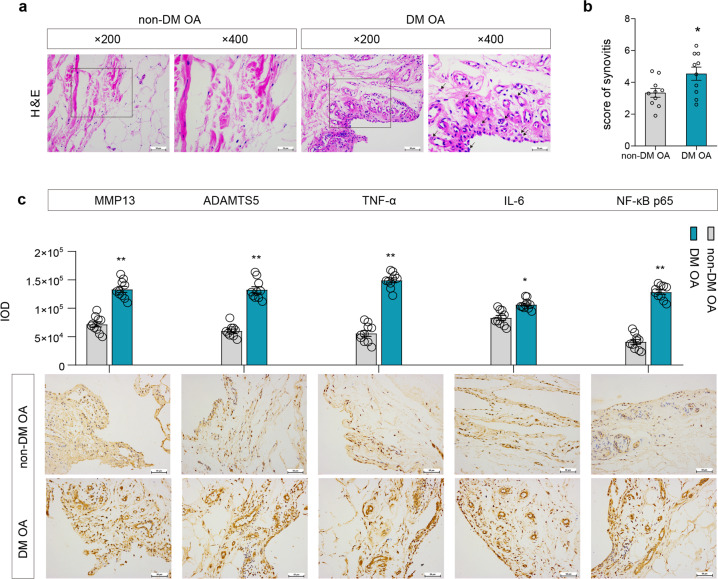
The morphology and the expression level of inflammatory and degradation factors in synovial tissue in patients with DM OA. **a**, **b** H&E staining of the synovial tissue of the non-DM OA or DM OA patients (**a**) and the synovitis score (**b**), ×200, ×400. **c** Representative IHC images and quantification of the integrated optical density (IOD) value of the inflammatory and degradation factors of synovial tissue in the non-DM OA and DM OA groups; scale bar: 50 μm. Ten fields of view for each sample were selected for analysis. The values are the means ± S.E.M., *n* = 10. The Mann–Whitney *U* test was used for statistical analysis. ^*^P < 0.05, ^**^P < 0.01 vs. non-DM OA.

In the synovial tissue of patients with knee OA, H&E staining showed that the synovial cell layer of OA patients with DM (DM OA) was thicker, the cell density of the matrix increased, and angiogenesis and inflammatory cells infiltrated significantly compared with the synovial tissue of OA patients without DM (non-DM OA) (Fig. 1a). The synovitis score results showed that there was a significant difference between the non-DM OA group and the DM OA group (Fig. 1b, *P* = 0.03). The IHC results showed that the protein expression levels of MMP13 and ADAMTS5 and the levels of the inflammatory factors TNF-α, IL-6, and NF-κB p65 in the synovium of the DM OA group were significantly higher than those of the non-DM OA group (Fig. 1c, *P* < 0.05, *P* < 0.05). The above results suggested that the synovium of the OA with DM group showed a more obvious inflammatory response than that of the OA alone group.

**Fig. 2:**
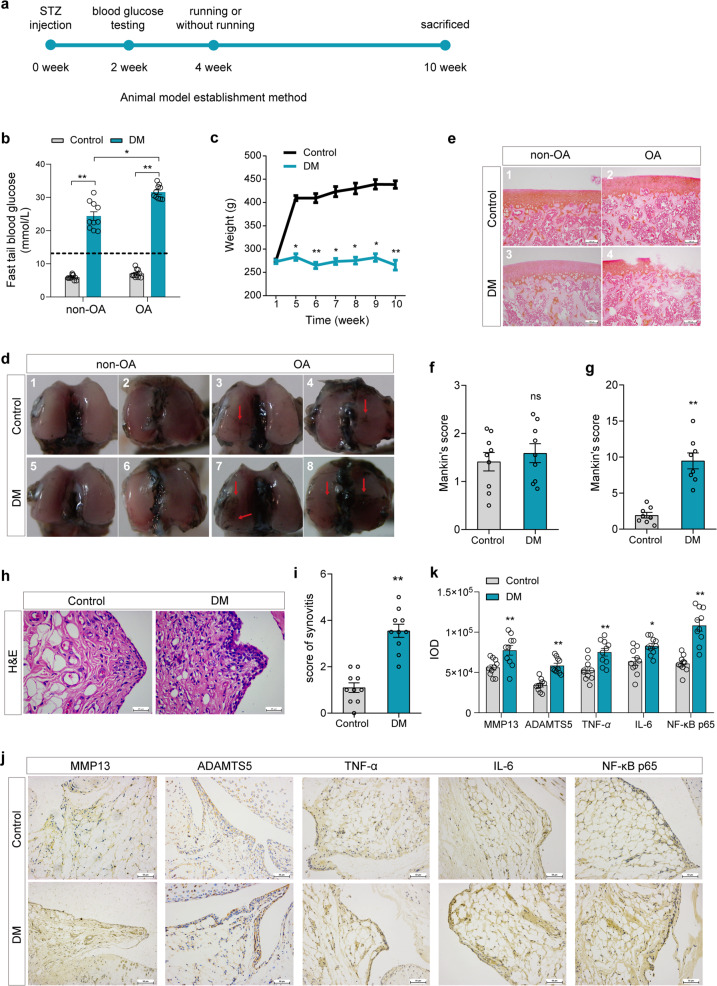
The establishment of a diabetic rat model and the expression level of inflammatory and degradation factors in synovial tissue. **a** Animal model establishment method. **b** The blood glucose levels of rats in the different groups. **c** The weight changes of rats in the different groups after injection of STZ at 1 week and 5 to 10 weeks. **d** India ink staining of the articular cartilage of the femoral condyle in rats in the different groups. **e–g** Safranin O staining (**e**) of tibial plateau cartilage of rats in the different groups, scale bar: 100 μm. **h, i**: H&E staining (**h**) and synovitis score (**i**) of the knee joint synovium of rats in the control and DM groups, scale bar: 25 μm. **j, k** Representative IHC images and the IOD values of inflammatory and degradation factors in synovial tissue in the control and DM groups; scale bar: 50 μm. Ten fields of view for each sample were selected for analysis. The values are the means±S.E.M., *n* = 10. The Mann–Whitney *U* test was used for statistical analysis. ^*^P < 0.05, ^**^P < 0.01 vs. the control.

In the rats with knee OA, the blood glucose results showed that the concentration of blood glucose in the DM group was higher than that in the control group and more than 20 mmol in both the nonrunning group and the running group (Fig. 2b, *P* < 0.01). In addition, the blood glucose level in the DM group increased significantly after running (Fig. 2b, *P* < 0.05). There was no significant difference in body weight between the DM group and the control group in the first week, but this value was significantly lower than that in the control group after the fifth week (Fig. 2c, *P* < 0.05, *P* < 0.01). The results of India ink staining on the articular surface of the knee showed that there was no significant difference in the general morphology of articular cartilage between the DM group and the control group (Fig. 2d1, d2, d5, d6). The surface of the articular cartilage was smooth and intact, and there was no fibrosis or ulceration. However, following long-distance running stimulation to induce OA, the cartilage surface of the tibial plateau was severely worn compared with that of the normal control group, with a rough cartilage surface and luster loss (Fig. 2d3,2d4, d7, d8).

Furthermore, the pathological changes in cartilage in the rats with DM with or without running-induced OA were tested by safranin O staining. The results showed that without running, the articular cartilage in both the control and DM groups manifested an intact cartilage structure, a regular cell arrangement, a smooth cartilage surface, uniform cartilage matrix staining, and a normal deep structure (Fig. 2e). No significant difference in Mankin’s score was found between the DM group and the control group (Fig. 2f, *P* > 0.05). When OA was induced by running, a slight staining of the cartilage matrix and slight roughness appeared in the control group, while defective cartilage surfaces, disordered cartilage matrix structures, thinner cartilage thicknesses, faded matrix staining, obviously reduced cartilage cells and forward tidal lines were found in the DM group (Fig. 2e, *P* < 0.01). The cartilage Mankin’s score of the DM group was significantly higher than that of the control group (Fig. 2g, *P* < 0.01).

Then, we observed histopathological changes in the synovium of the rats with DM. Compared with that of the normal control group, H&E staining (Fig. 2h) of knee joint synovium in the DM group showed thickened lining of cell layers, increased interstitial cells and infiltrated inflammatory cells, and a significantly increased synovitis histology score (4.00 ± 0.82) (Fig. 2i, *P* < 0.01). The IHC results showed that the protein expression levels of MMP-13, ADAMTS5, TNF-α, IL-6, and NF-κB p65 were significantly increased in the knee joint synovium of the rats in the DM group (Fig. 2j, k, *P* < 0.05, *P* < 0.01). Moreover, compared with that of the normal control group, the expression of matrix synthesis-related protein (COL2A1) was decreased, while the levels of matrix degradation and inflammatory markers (MMP13, ADAMTS5, TNF-α, IL-6) were increased in the cartilage in the DM group (Supplementary Fig. S1, *P* < 0.05, *P* < 0.01).

These results suggested that chronic hyperglycemia in DM was able to promote the expression of degradation and inflammatory factors in the knee joint synovium of rats, induce moderate synovitis, cause matrix degradation and inflammation in cartilage, and induce OA under normal diet/running stimulation.

### FLSs stimulated by high glucose can release inflammatory factors and cause chondrocyte injury

**Fig. 3:**
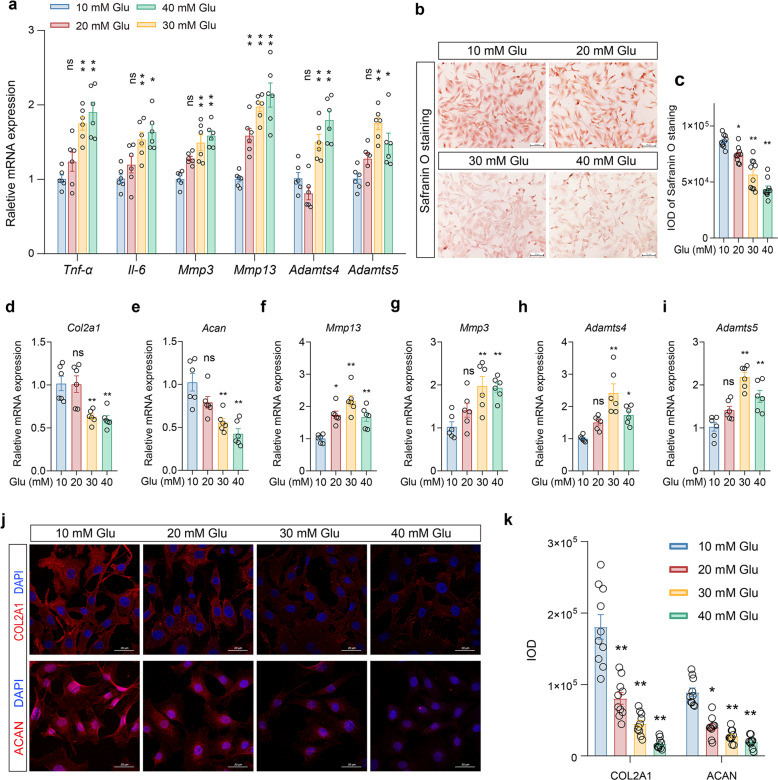
The effect of different concentrations of glucose-stimulated FLSs on chondrocytes. **a** After FLSs were treated with different concentrations of glucose (Glu) for 3 days, the mRNA expression of inflammatory and matrix degradation-related factors in FLSs was detected. **b–i** Chondrocytes were cocultured with FLSs with different concentrations of glucose for 3 days through the Transwell experiment. Chondrocytes were stained with safranin O dye (**b**), and the IOD (**c**) was calculated. The mRNA expression of matrix synthesis- and degradation-related genes was detected by RT-qPCR (**d-i**). **j, k** IF detection by confocal microscopy of COL2A1 and ACAN expression (**j**) in chondrocytes and its IOD value (**k**). Ten fields of view for each IF sample were selected for analysis. The values are the means±S.E.M., *n* = 6. One-way ANOVA was used for statistical analysis. ^*^P < 0.05, ^**^P < 0.01 vs. the 10 mM Glu (control) group. Scale bar: 20 μm.

To explore whether the changes in cartilage and synovium induced by the DM environment account for hyperglycemia, we performed in vitro experiments. First, FLSs from rats were treated with different concentrations of glucose (10, 20, 30, 40 mM Glu) for 3 days, and the mRNA expression of inflammatory- and degradation-related factors was detected by RT-qPCR. The results showed that at 20 mM Glu, the mRNA expression of the degradation-related gene *Mmp13* began to increase significantly, while the mRNA expression of other inflammatory degradation factors (*Tnf-α, Il-6, Mmp3, Adamts4, and Adamts5*) did not change significantly (Fig. 3a, *P* < 0.01). In the presence of 30 mM Glu and 40 mM Glu, the mRNA expression levels of these inflammatory and degradation factors were significantly increased (Fig. 3a, *P* < 0.01, *P* < 0.05).

Furthermore, FLSs treated with different concentrations of glucose were cocultured with chondrocytes for 3 days via a Transwell experiment, and the effect of FLSs on chondrocytes was detected. The results of safranin O staining and IOD statistical analysis showed that compared with that of the control group (10 mM Glu), chondrocyte staining began to fade when the concentration of glucose was higher than 20 mM (Fig. 3b, c, *P* < 0.01, *P* < 0.05), and the chondrocyte count decreased slightly at 40 mM. Moreover, with 30 mM Glu and 40 mM Glu, the mRNA expression of genes related to matrix synthesis (*Col2a1* and *Acan*) decreased significantly (Fig. 3d, e, *P* < 0.01), while the expression of matrix degradation-related genes (*Mmp13, Mmp3, Adamts4*, *and Adamts5*) increased significantly (Fig. 3f–i, *P* < 0.01). IF and IOD statistical analysis showed that when the concentration of glucose was 20 mM or higher, the expression of matrix-related proteins (COL2A1 and ACAN) in chondrocytes decreased significantly (Fig. 3j, k, *P* < 0.01, *P* < 0.05).

The above results suggested that FLSs stimulated by high glucose can reduce matrix synthesis and enhance the degradation of chondrocytes by promoting the expression and release of matrix-degrading enzymes and inflammatory factors.

### Hyperglycemia induced AGEs accumulation and inflammation in FLSs through the HIF-1α/GLUT1 pathway

**Fig. 4:**
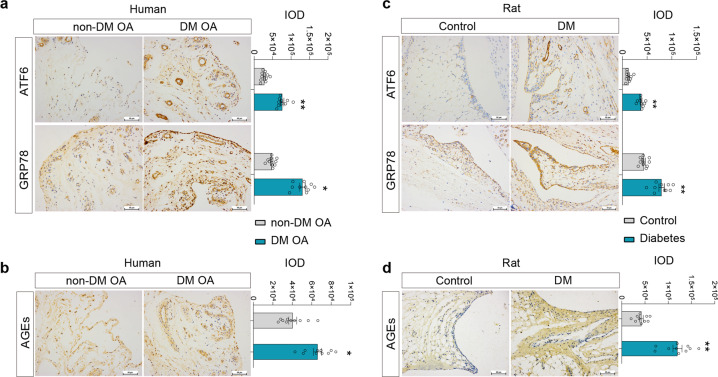
The level of ERS and the content of AGEs of the patients with DM OA and the rats with diabetes. **a, b** Representative IHC images and the IOD values of GRP78 and ATF6 (**a**) and AGEs (**b**) in the synovial tissue of the patients in the DM OA or the non-DM OA group. **c, d** Representative IHC images and the IOD values of GRP78 and ATF6 (**c**) and AGEs (**d**) in the synovial tissue of the rats in the control or DM group. Ten fields of view for each sample were selected for analysis. The values are the means±S.E.M., human/rat: *n* = 10. The Mann–Whitney *U* test was used for statistical analysis. ^*^P < 0.05, ^**^P < 0.01 vs. non-DM OA or control. Scale bar: 50 μm.

Based on the strong correlation between synovitis and the occurrence of OA^7,27^, synovitis in the DM environment may also have a potential contribution to the occurrence of DM OA. Therefore, we further explored the possible mechanism of synovitis caused by DM. Studies have shown that the ERS level and the content of AGEs play a significant role in inducing the production of inflammatory cytokines, especially in the tissue injury caused by DM^28,29^. The level of ERS in synovial tissue of patients with DM OA was detected by IHC. The results showed that the expression of the ERS-related proteins ATF6 and GRP78 in the DM OA group was significantly higher than that in the non-DM OA group (Fig. 4a, *P* < 0.05, *P* < 0.01), and the content of AGEs was also significantly increased (Fig. 4b, *P* < 0.05). Similarly, we further verified in the DM rat model that the expression of ATF6 and GRP78 and the content of AGEs in the synovium of the DM group were significantly increased compared with those of the control group (Fig. 4c, d, *P* < 0.05, *P* < 0.01). Then, after FLSs were treated with 100 ng/ml exogenous AGE-BSA for 3 days, we found that the mRNA expression of inflammatory degradation-related factors was significantly increased compared with that of the control group (Supplementary Fig. S2a, *P* < 0.01, *P* < 0.05), which was similar to the effect of 30 mM Glu treatment. Furthermore, combined with 1 IU/ml AGEs, the receptor inhibitor LMWH inhibited the increased expression of inflammatory degradation factors induced by exogenous AGEs or 30 mM high glucose (Supplementary Fig. S2a, *P* < 0.01, *P* < 0.05). Similarly, treatment with the ERS agonist Ti at 1 μg/ml for 3 days could induce the expression of inflammatory degradation factors in FLSs, which was consistent with the results of 30 mM high glucose treatment, while combination with the ERS inhibitor 4-PBA at 250 μm could inhibit the increased expression of inflammatory degradation factors induced by Ti or 30 mM high glucose (Supplementary Fig. S2b, *P* < 0.01, *P* < 0.05). These results suggested that hyperglycemia could increase the level of ERS and the accumulation of AGEs in the joint synovium and then induce synovitis.

**Fig. 5:**
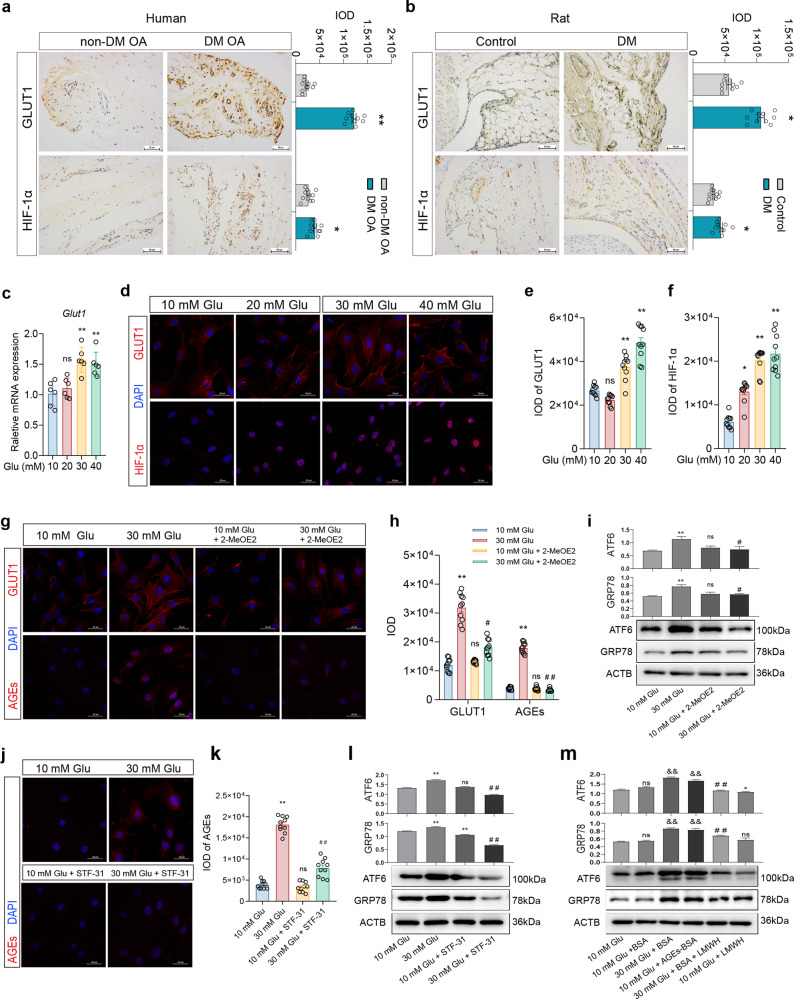
The effect of a high concentration of glucose on FLSs. **a** Representative IHC images and the IOD value of HIF-1α and GLUT1 in the synovium of the patients (**a**) and of the rats **(b)**, scale bar: 50 μm. **c** The mRNA expression of *Glut1* in the FLSs treated with different concentrations of glucose. **d-h** IF staining and the IOD values of GLUT1, HIF-1α and AGEs in the FLSs; scale bar: 20 μm. **i, l, m** Protein expression of GRP78 and ATF6 of FLSs. **j, k:** IF staining and the IOD value of AGEs of the FLSs, scale bar: 20 μm. Ten fields of view for each IF sample were selected for analysis. The values are the means ± S.E.M., human/rat: n = 10, cellular experiments: *n* = 6. Statistical analysis: Mann–Whitney *U* test for (**a, b**), ^*^P < 0.05, ^**^P < 0.01 vs. non-DM OA or control; one-way ANOVA for (**c–f**), unpaired Student’s *t*-test for (**g**)-(**m**). ^*^P < 0.05, ^**^P < 0.01 vs. the 10 mM Glu (control) group. ^&^*P* < 0.05, ^&&^*P* < 0.01 vs. the 10 mM Glu+BSA group. ^#^*P* < 0.05, ^##^*P* < 0.01 vs. the 30 mM Glu group or the 30 mM Glu+BSA group.

Studies have shown that AGEs are the products of excess sugar and protein binding, so we hypothesized that GLUT1 may play a critical role in glucose transport of FLSs in DM. The results showed that the expression level of GLUT1 protein in the synovium of the DM OA group was significantly higher than that of the non-DM OA group (Fig. 5a, *P* < 0.01). Similarly, in the DM rat model, the expression of GLUT1 protein in the synovium of the DM group was significantly higher than that of the control group (Fig. 5b, *P* < 0.05). Hyperglycemia can mediate diabetic nephropathy by inducing an increase in HIF-1α expression and regulating the expression of downstream GLUT1^30^. In this study, we found that the level of HIF-1α protein in human synovial tissue of the DM OA group and that of rats with DM was significantly higher than that in the non-DM group (Fig. 5a, b, *P* < 0.05). These results suggested that the DM environment could induce the increased expression of GLUT1 and HIF-1α.

Furthermore, we verified that high glucose induced an increase in ERS levels and AGEs accumulation in FLSs via the HIF-1α/GLUT1 pathway in vitro. The results showed that after FLSs were treated with different concentrations of glucose for 3 days, the mRNA and protein expression levels of GLUT1 were significantly increased in the 30 mM Glu and 40 mM Glu groups compared with the control group (Fig. 5c, e, *P* < 0.01). Moreover, nuclear staining of HIF-1α was enhanced in the cells treated with 20 mM or higher Glu (Fig. 5d, f, *P* < 0.01, *P* < 0.05). Furthermore, we found that compared with that of the control group, the content of AGEs increased significantly under 30 mM Glu treatment (Fig. 5g, h, *P* < 0.01), and the mRNA expression of the inflammatory factors *Tnf-a* and *Il-6* and the degradation factors *Mmp3*, *Mmp13*, *Adamts4*, and *Adamts5* increased (Supplementary Fig. S2a, *P* < 0.01, *P* < 0.05). After the activity of HIF-1α was inhibited by 2.5 μM of the HIF-1α inhibitor 2-MeOE2 under 30 mM Glu, the protein expression level and AGEs content of GLUT1 were significantly lower than those of 30 mM Glu group (Fig. 5g, h, *P* < 0.01, *P* < 0.05), and the mRNA expression of inflammatory degradation factors was also significantly decreased (Supplementary Fig. S2c, *P* < 0.01, *P* < 0.05). Moreover, the Western blot results showed that 30 mM Glu remarkably increased the expression of the ERS-related proteins GPR78 and ATF6, which were was inhibited by 2-MeOE2, compared with that of the control group (Fig. 5i, *P* < 0.01, *P* < 0.05). We further found that the GLUT1 inhibitor STF-31 at 0.5 μM could significantly inhibit the increase in AGEs content (Fig. 5j, k, *P* < 0.01), the increase in ERS levels (Fig. 5l, *P* < 0.01) and the mRNA expression of inflammatory degradation factors (Supplementary Fig. S2d, *P* < 0.01, *P* < 0.05) induced by 30 mM Glu. Then, after FLSs were treated with 100 ng/ml exogenous AGE-BSA for 3 days, the expression of ERS-related proteins was significantly enhanced (Fig. 5m, *P* < 0.01, *P* < 0.05), which was similar to that of the 30 mM Glu treatment. When combined with 1 IU/ml AGEs, the receptor inhibitor LMWH inhibited the increase in ERS levels induced by exogenous AGEs or 30 mM high glucose (Fig. 5m, *P* < 0.01, *P* < 0.05).

The above results suggested that high glucose could induce the nuclear expression of HIF-1α in FLSs and promote the high expression of GLUT1, which leads to the accumulation of AGEs and the activation of ERS, contributing to the increased expression of inflammatory degradation factors.

### Inhibition of GLUT1 and HIF-1α expression in FLSs under high glucose stimulation can reverse its effect on chondrocytes

**Fig. 6:**
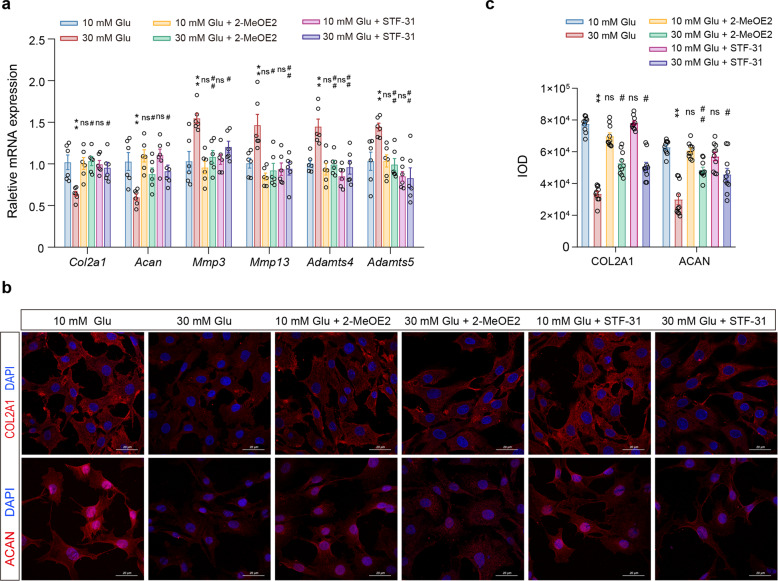
The effect of FLSs on chondrocytes after inhibiting the expression of GLUT1 and HIF-1α under high glucose stimulation. **a–c** Chondrocytes were cocultured with FLSs for 3 days through Transwell experiments with different treatments. RT-qPCR was used to detect the mRNA expression of *Col2a1*, *Acan*, *Mmp3*, *Mmp13*, *Adamts4*, and *Adamts5* (**a**). IF staining of COL2A1 and ACAN (**b**) and its quantification of the IOD value (**c**), scale bar: 20 μm. Ten fields of view for each IF sample were selected for analysis. The values are the means±S.E.M., n = 6. An unpaired Student’s *t*-test was used for statistical analysis. ^*^P < 0.05, ^**^P < 0.01 vs. the 10 mM Glu (control) group. ^#^*P* < 0.05, ^##^*P* < 0.01 vs. the 30 mM Glu group.

Finally, we used a Transwell experiment to further explore the effect of inhibiting the expression of HIF-1α and GLUT1 in FLSs under high glucose stimulation on chondrocytes. The experiment was divided into 6 groups: normal control group (10 mM Glu), 30 mM Glu treatment group, 30 mM Glu plus 2-MeOE2 group, 30 mM Glu plus STF-31 group, 2-MeOE2 treatment group, and STF-31 treatment group. The results showed that the FLSs treated with 2-MeOE2 and STF-31 alone showed no significant change in the mRNA expression of matrix synthesis and degradation genes in chondrocytes, but compared with that of the 30 mM Glu treatment group, the mRNA expression of the matrix marker genes *Col2a1* and *Acan* increased significantly in the chondrocytes treated with 30 mM Glu plus 2-MeOE2 or 30 mM Glu plus STF-31, while the expression of the degradation-related genes *Mmp3*, *Mmp13*, *Adamts4* and *Adamts5* decreased significantly (Fig. 6a, *P* < 0.01, *P* < 0.05). The results of IF staining and IOD analysis showed that 2-MeOE2 or STF-31 could significantly attenuate the inhibitory effect of the FLSs stimulated with 30 mM Glu on the expression of COL2A1 and ACAN in chondrocytes (Fig. 6b, c, *P* < 0.01, *P* < 0.05).

These results suggested that high glucose can promote the inflammatory and degradation reactions of chondrocytes and inhibit matrix synthesis by inducing the increased expression of HIF-1α and GLUT1 in FLSs.

## Discussion

### DM aggravates the progression of knee OA

Both epidemiological investigations and experimental studies have indicated the correlation of DM and OA^6,11,31,32^. However, how DM affects the progression of OA is not clear, as other confounding factors, such as age, sex, obesity, and some additional metabolic factors, are also considered risk factors for OA^4^. In the current study, according to a previous method, we established an animal model of DM, and the results showed that the rats in the DM group were continuously exposed to hyperglycemia, but their body weight was significantly decreased, which was related to the decrease in insulin secretion induced by STZ. Then, we investigated the adverse effects of hyperglycemia on the progression of knee OA and found that the inflammatory score of the synovium from the OA patients with DM was much higher than that of the OA patients without DM. Such findings were further confirmed in rats with STZ-induced DM undergoing OA modeling via excessive running. Interestingly, the results showed that the rats with DM subjected to excessive running exhibited higher serum glucose than the rats with DM not subjected to running, which was accompanied by an aggravated inflammatory reaction of the synovium and degenerative features of the cartilage. Such findings indicated that DM might aggravate the progression of knee OA.

### Hyperglycemia stimulated the inflammatory reaction of the synovium

Synovitis is one of the typical features and essential regulators of OA progression. Inflammatory factors, as well as MMP and ADAMTS proteases, are released from the synovium, which further accelerates the progression of OA^33,34^. Synovial tissue is rich in blood vessels, while articular cartilage lacks blood supply, so the synovium is more likely to be stimulated by hyperglycemia. Previous studies have revealed that FLSs are much more sensitive to hyperglycemia than chondrocytes^35^. FLSs not only secrete inflammatory factors, including TNFs and ILs, into the articular cavity^36^ but also release more MMPs and ADAMTSs after being stimulated by inflammation in the joint^7^, which further induces inflammation and degeneration of articular cartilage^14,37,38^. The clinical data from Luo et al. showed that the concentration of MMPs in the synovial fluid of patients in the DM-OA group was significantly higher than that in the OA group without DM or the healthy control group^39^. Other studies also showed that DM patients had significantly higher concentrations of IL-6 and IL-1β in the synovial fluid than non-DM patients^40,41^. These data suggest that DM leads to increased levels of ECM degenerative enzymes and inflammatory factors in synovial fluid. In our current study, an enhanced inflammatory reaction was observed in the synovium from the OA with DM group, both in humans and in rats.

It has been reported that glucose is one of the differentially abundant metabolites in the synovial fluid between DM and non-DM patients^42^. To date, most studies have focused on the direct adverse effect of high glucose on cartilage. However, the synovium is a joint tissue with an abundant blood supply; thus, it is directly exposed to high blood glucose, while the blood glucose components in the synovial fluid are also filtered through the synovium^7^, which indicates that the indirect effect of synovium on the cartilage in a high glucose environment caused by DM cannot be neglected. In this study, we explored a novel mechanism by which hyperglycemia affects cartilage. Our in vitro results confirmed that excessive glucose could induce a degenerative phenotype of chondrocytes through FLSs. Thus, we believe that hyperglycemia induces or aggravates the degeneration of the articular cartilage by stimulating the inflammatory reaction of the synovium.

### Accumulation of AGEs induced by high glucose stimulated the release of inflammatory factors from FLSs

ERS is often considered one of the initial reactions of cellular inflammation^43,44^. AGEs are proteins or lipids that become glycated as a result of exposure to glucose. RAGE is the receptor of AGEs, which triggers ERS and downstream inflammatory signaling after being stimulated by AGEs^45,46^ and therefore participates in multiple degenerative diseases, such as diabetes, chronic kidney disease, and Alzheimer’s disease^20,28,29^. In the present study, AGEs content, ERS levels, and inflammatory reactions were all stimulated in both the human and rat synovium with OA and DM compared with those with OA alone. Furthermore, inflammatory factors secreted by FLSs were also stimulated by 30 mM glucose or the ERS agonist Ti, while the ERS inhibitor 4-PBA notably attenuated the proinflammatory effects of high glucose on FLSs in vitro. Moreover, high glucose and AGE-BSA could both induce the expression of GRP78 and ATF6, as well as MMPs and ADAMTSs, while LMWH, a RAGE inhibitor, could block the proinflammatory effects of high glucose. This evidence indicated that high glucose induced the accumulation of AGEs in the FLSs, which further activated RAGE and stimulated the ERS and expression of inflammatory factors in the FLSs.

### Activation of HIF-1α/GLUT1 signaling participated in the accumulation of AGEs induced by high glucose

GLUT1 is an essential glucose transporter that maintains the fuel supply of almost all cell types^15^. Stimulation of GLUT1 increases the influx of glucose to the cytoplasm, which is involved in the Maillard reaction with some lipids or proteins. This reaction produces excessive intracellular AGEs and results in oxidative damage and subsequent glomerular sclerosis and retinopathy in DM patients^16,21,45^. Other studies found that GLUT1 presented a more critical role in the degenerative changes in DM patients than any other subtype of GLUTs^47,48^. Here, we also found that the GLUT1 level in patients or rats with both DM and OA was higher than that in rats with OA only. In addition, the expression of GLUT1 was stimulated by high glucose in FLSs in vitro, which was accompanied by increased levels of AGEs, ERS, and inflammatory factors. STF-31, a GLUT1-specific inhibitor, could significantly attenuate such changes in FLSs and finally relieve the degeneration of cocultured chondrocytes in the Transwell system. These findings indicated a critical role of GLUT1 in the proinflammatory effects of high glucose on FLSs. As inhibiting GLUT1 might attenuate RA progression^49^, as well as the deterioration of some cancers^50^, synovial GLUT1 might be a potential target for the treatment of knee OA.

HIF-1α is a critical trans-regulator in glucose transportation, angiogenesis, and cell metabolism and therefore helps cells survive under hypoxia^51^. Other metabolic or inflammatory factors, including AGEs, also regulate the function of HIF-1α^52^. Recent reports have indicated a key role of HIF-1α in DM-related disorders^30,53^. Stimulated HIF-1α expression in the synovium was observed in both humans and rats with DM and OA compared with those with OA only. Additionally, the HIF-1α inhibitor 2-MeOE2 attenuated the effect of high glucose stimulation on GLUT1 expression, AGEs accumulation, and inflammatory gene expression in FLSs, which further resulted in attenuated degenerative features of cocultured chondrocytes in the Transwell system. Interestingly, studies also revealed that activation of RAGE by AGEs could stimulate HIF-1α signaling in multiple tissues^54^. Therefore, we inferred that there might be a HIF-1α-GLUT1-AGEs-HIF-1α loop in DM-induced synovitis, which aggravates the progression of OA.

In the future, however, we still need to further confirm the therapeutic targets of DM OA through animal experiments and clinical studies. Moreover, the safety and effectiveness of related intervention drugs need to be considered.

**Fig. 7:**
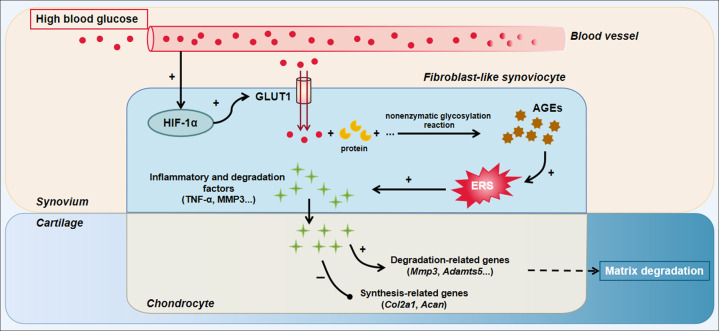
Summary diagram. High glucose-induced cartilage inflammation and degeneration by stimulating AGEs accumulation in FLSs through HIF-1α-GLUT1-AGEs signaling.

In the current study, we revealed that high glucose-stimulated HIF-1α-GLUT1-AGEs signaling in FLSs, which subsequently activated ERS and the release of proinflammatory factors from the synovium and finally induced inflammation and degeneration of articular cartilage (Fig. 7). These findings provide a novel view of the relationship between DM and OA, as well as the pathogenesis and treatment of OA.

## Supplementary information

Supplemental material

## References

[1] Xie F (2016). Economic and humanistic burden of osteoarthritis: a systematic review of large sample studies. Pharmacoeconomics.

[2] Sharma L, Kapoor D, Issa S (2006). Epidemiology of osteoarthritis: an update. Curr. Opin. Rheumatol..

[3] Saeedi P (2019). Global and regional diabetes prevalence estimates for 2019 and projections for 2030 and 2045: Results from the International Diabetes Federation Diabetes Atlas, 9(th) edition. Diabetes Res. Clin. Pract..

[4] Berenbaum F (2011). Diabetes-induced osteoarthritis: from a new paradigm to a new phenotype. Ann. Rheum. Dis..

[5] Neumann J (2019). Diabetics show accelerated progression of knee cartilage and meniscal lesions: data from the osteoarthritis initiative. Skelet. Radio..

[6] Kelley KM, Russell SM, Matteucci ML, Nicoll CS (1993). An insulin-like growth factor I-resistant state in cartilage of diabetic rats is ameliorated by hypophysectomy. Possible role of metabolism. Diabetes.

[7] Scanzello CR, Goldring SR (2012). The role of synovitis in osteoarthritis pathogenesis. Bone.

[8] Wang T, He C (2018). Pro-inflammatory cytokines: the link between obesity and osteoarthritis. Cytokine Growth Factor Rev..

[9] Chen B, Li Y, Liu Y, Xu Z (2019). circLRP6 regulates high glucose-induced proliferation, oxidative stress, ECM accumulation, and inflammation in mesangial cells. J. Cell. Physiol..

[10] Zhang Y (2017). Protection of Mcc950 against high-glucose-induced human retinal endothelial cell dysfunction. Cell Death Dis..

[11] Atayde SA (2012). Experimental diabetes modulates collagen remodelling of joints in rats. Histol. Histopathol..

[12] Zhang W (2017). Hyperglycemia-related advanced glycation end-products is associated with the altered phosphatidylcholine metabolism in osteoarthritis patients with diabetes. PloS One.

[13] Liang H (2019). Toll-like receptor 4 promotes high glucose-induced catabolic and inflammatory responses in chondrocytes in an NF-kappaB-dependent manner. Life Sci..

[14] Mathiessen A, Conaghan PG (2017). Synovitis in osteoarthritis: current understanding with therapeutic implications. Arthritis Res. Ther..

[15] Holman GD (2020). Structure, function and regulation of mammalian glucose transporters of the SLC2 family. Pflug. Arch..

[16] Heilig CW (2013). GLUT1 regulation of the pro-sclerotic mediators of diabetic nephropathy. Am. J. Nephrol..

[17] Petrasca A (2020). Targeting bioenergetics prevents CD4 T cell-mediated activation of synovial fibroblasts in rheumatoid arthritis. Rheumatol. (Oxf.).

[18] Garcia-Carbonell R (2016). Critical role of glucose metabolism in rheumatoid arthritis fibroblast-like synoviocytes. Arthritis Rheumatol..

[19] Qing-Xian L (2020). Programming changes in GLUT1 mediated the accumulation of AGEs and matrix degradation in the articular cartilage of female adult rats after prenatal caffeine exposure. Pharmacol. Res..

[20] Suzuki R (2020). Intracellular accumulation of advanced glycation end products induces osteoblast apoptosis via endoplasmic reticulum stress. J. Bone Miner. Res..

[21] Rasheed Z, Akhtar N, Haqqi TM (2011). Advanced glycation end products induce the expression of interleukin-6 and interleukin-8 by receptor for advanced glycation end product-mediated activation of mitogen-activated protein kinases and nuclear factor-kappaB in human osteoarthritis chondrocytes. Rheumatol. (Oxf.).

[22] Ku PM (2011). Molecular role of GATA binding protein 4 (GATA-4) in hyperglycemia-induced reduction of cardiac contractility. Cardiovasc. Diabetol..

[23] Liu F, Xie M, Chen D, Li J, Ding W (2013). Effect of VIVO(dipic-Cl)(H2O)2 on lipid metabolism disorders in the liver of STZ-induced diabetic rats. J. Diabetes Res..

[24] Tang T (2008). Serum keratan sulfate transiently increases in the early stage of osteoarthritis during strenuous running of rats: protective effect of intraarticular hyaluronan injection. Arthritis Res. Ther..

[25] van der Sluijs JA (1992). The reliability of the Mankin score for osteoarthritis. J. Orthop. Res..

[26] Krenn V (2006). Synovitis score: discrimination between chronic low-grade and high-grade synovitis. Histopathology.

[27] Loeuille D (2009). Magnetic resonance imaging in osteoarthritis: which method best reflects synovial membrane inflammation? Correlations with clinical, macroscopic and microscopic features. Osteoarthr. Cartil..

[28] Gowd V (2020). Resveratrol: evidence for its nephroprotective effect in diabetic nephropathy. Adv. Nutr..

[29] Liao Z (2019). Exosomes from mesenchymal stem cells modulate endoplasmic reticulum stress to protect against nucleus pulposus cell death and ameliorate intervertebral disc degeneration in vivo. Theranostics.

[30] Nayak BK (2016). HIF-1 mediates renal fibrosis in OVE26 type 1 diabetic mice. Diabetes.

[31] Sturmer T, Brenner H, Brenner RE, Gunther KP (2001). Non-insulin dependent diabetes mellitus (NIDDM) and patterns of osteoarthritis. The Ulm osteoarthritis study. Scand. J. Rheumatol..

[32] Puenpatom RA, Victor TW (2009). Increased prevalence of metabolic syndrome in individuals with osteoarthritis: an analysis of NHANES III data. Postgrad. Med..

[33] Sowers M, Karvonen-Gutierrez CA, Jacobson JA, Jiang Y, Yosef M (2011). Associations of anatomical measures from MRI with radiographically defined knee osteoarthritis score, pain, and physical functioning. J. Bone Jt. Surg. Am..

[34] Stafford CT, Niedermeier W, Holley HL, Pigman W (1964). Studies on the concentration and intrinsic viscosity of hyaluronic acid in synovial fluids of patients with rheumatic diseases. Ann. Rheum. Dis..

[35] Gui T (2019). Elevated expression of ICAM-1 in synovium is associated with early inflammatory response for cartilage degeneration in type 2 diabetes mellitus. J. Cell. Biochem..

[36] Sellam J, Berenbaum F (2010). The role of synovitis in pathophysiology and clinical symptoms of osteoarthritis. Nat. Rev. Rheumatol..

[37] Sutton S (2009). The contribution of the synovium, synovial derived inflammatory cytokines and neuropeptides to the pathogenesis of osteoarthritis. Vet. J..

[38] Stone AV (2014). Pro-inflammatory stimulation of meniscus cells increases production of matrix metalloproteinases and additional catabolic factors involved in osteoarthritis pathogenesis. Osteoarthr. Cartil..

[39] Luo S (2019). Expression and significance of MMPs in synovial fluid, serum and PBMC culture supernatant stimulated by LPS in osteoarthritis patients with or without diabetes. Exp. Clin. Endocrinol. Diabetes.

[40] Siu KK (2013). Increased interleukin 1beta levels in the subacromial fluid in diabetic patients with rotator cuff lesions compared with nondiabetic patients. J. Shoulder Elb. Surg..

[41] Eitner A (2017). Pain sensation in human osteoarthritic knee joints is strongly enhanced by diabetes mellitus. Pain.

[42] Zhang W (2016). Metabolomic analysis of human synovial fluid and plasma reveals that phosphatidylcholine metabolism is associated with both osteoarthritis and diabetes mellitus. Metabolomics.

[43] Di Prisco GV, Huang W, Buffington SA (2014). Translational control of mGluR-dependent long-term depression and object-place learning by eIF2alpha. Nat. Neurosci..

[44] Way SW, Popko B (2016). Harnessing the integrated stress response for the treatment of multiple sclerosis. Lancet Neurol..

[45] Perrone A, Giovino A, Benny J, Martinelli F (2020). Advanced glycation end products (AGEs): biochemistry, signaling, analytical methods, and epigenetic effects. Oxid. Med. Cell. Longev..

[46] Dariya B, Nagaraju GP (2020). Advanced glycation end products in diabetes, cancer and phytochemical therapy. Drug Discov. Today.

[47] Vignali D (2018). Detection and characterization of CD8(+) autoreactive memory stem T cells in patients with type 1 diabetes. Diabetes.

[48] Bolla AM (2020). Expression of glucose transporters in duodenal mucosa of patients with type 1 diabetes. Acta Diabetol..

[49] Gallagher L (2020). Insulin-resistant pathways are associated with disease activity in rheumatoid arthritis and are subject to disease modification through metabolic reprogramming: a potential novel therapeutic approach. Arthritis Rheumatol..

[50] Meng Y (2019). The progress and development of GLUT1 inhibitors targeting cancer energy metabolism. Future Med. Chem..

[51] Gonzalez FJ, Xie C, Jiang C (2018). The role of hypoxia-inducible factors in metabolic diseases. Nat. Rev. Endocrinol..

[52] Treins C, Giorgetti-Peraldi S, Murdaca J, Van Obberghen E (2001). Regulation of vascular endothelial growth factor expression by advanced glycation end products. J. Biol. Chem..

[53] Isoe T (2010). High glucose activates HIF-1-mediated signal transduction in glomerular mesangial cells through a carbohydrate response element binding protein. Kidney Int..

[54] Khan MI, Rath S, Adhami VM, Mukhtar H (2018). Hypoxia driven glycation: Mechanisms and therapeutic opportunities. Semin. Cancer Biol..

