# Author Correction: Hyperglycemia-induced accumulation of advanced glycosylation end products in fibroblast-like synoviocytes promotes knee osteoarthritis

**DOI:** 10.1038/s12276-022-00788-y

**Published:** 2022-06-15

**Authors:** Qingxian Li, Yinxian Wen, Linlong Wang, Biao Chen, Jun Chen, Hui Wang, Liaobin Chen

**Affiliations:** 1 Division of Joint Surgery and Sports Medicine, Department of Orthopedic Surgery, Zhongnan Hospital of Wuhan University, Wuhan, 430071 China; 2 Hubei Provincial Key Laboratory of Developmentally Originated Disease, Wuhan, 430071 China; 3 Joint Disease Research Center of Wuhan University, Wuhan, 430071 China; 4 Department of Pharmacology, School of Basic Medical Sciences, Wuhan University, Wuhan, 430071 China

**Keywords:** Glycobiology, Mechanisms of disease, Glycobiology, Mechanisms of disease

Correction to: *Experimental & Molecular Medicine* 10.1038/s12276-021-00697-6, published online 10 November 2021

The authors regret to notice that the representative Safranin O images of Fig. 2e1 and Fig. 2e3 were inadvertently duplicated during figure assembly. The Fig. 2e1 has been updated with a correct image. The authors believe that the corrections would not affect any results, discussions and conclusions displayed in the rest of the article. The authors would like to apologize for any inconvenience caused.

**Figures in the original manuscript:**

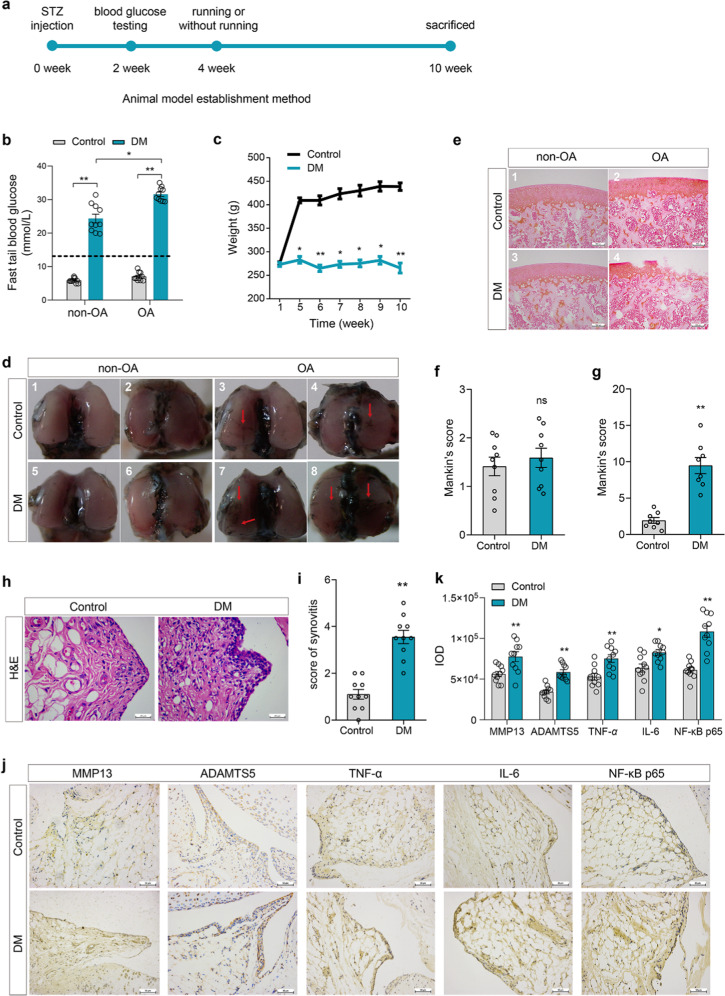



**Figures after the changes:**

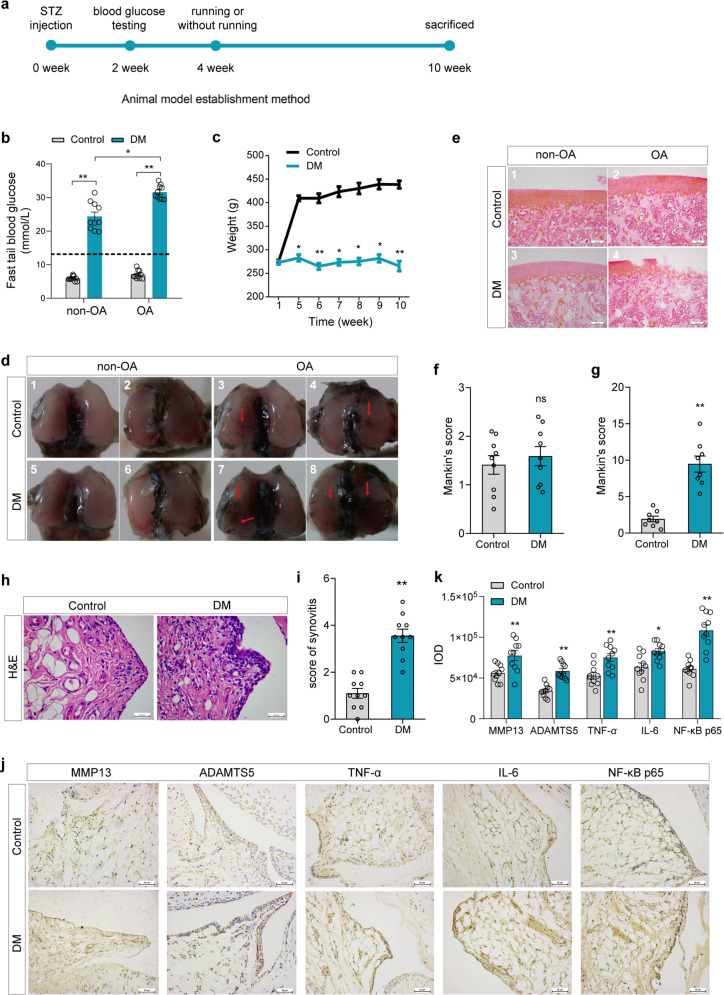



The original article has been corrected.

