# Supplementary material for: Hyperglycemia-induced accumulation of advanced glycosylation end products in fibroblast-like synoviocytes promotes knee osteoarthritis
Source: Exp Mol Med. 2021 Nov 10;53(11):1735–47. doi: 10.1038/s12276-021-00697-6 (PMC8639977; doi:10.1038/s12276-021-00697-6)
Supplement: Supplementary file 1 — Supplemental material [file 12276_2021_697_MOESM1_ESM.pdf]

SUPPLEMENTARY MATERIALS

1 Chemicals and reagents

D-Glucose (CAS No. 50-99-7) was obtained from Sinopharm Chemical Reagent Co., Ltd. (Shanghai, China). STZ (CAS No. S0130) was obtained from Sigma-Aldrich Co., Ltd. (St Louis, MO, USA). Isoflurane was purchased from Baxter Healthcare Co. (Deerfield, IL, USA). A TRIzol (SKU: R6830-02) kit was purchased from Invitrogen (Carlsbad, CA, USA). Reverse transcription and quantitative PCR (Q-PCR) kits were purchased from Takara Biotechnology Co., Ltd. (Dalian, China). Oligonucleotide primers for the gene used for analysis were synthesized by Sangon Biotech Co., Ltd. (Shanghai, China). SYBR Green dye was obtained from Applied Biosystems, by Thermo Fisher Scientific (ABI, Foster City, CA, USA). Hematoxylin (No. H8070) was purchased from Solarbio Science & Technology (Beijing, China), and eosin (No. E607321) was purchased from Sangon (Shanghai, China). Safranin O (CAS No. 477-73-6) was obtained from Hengyuan Biotech (Shanghai, China). Triton X100 (No. T8532) was purchased from Sigma-Aldrich (St. Louis, MO, USA). RIPA lysis buffer and a BCA Protein Assay kit were purchased from Beyotime Institute of Biotechnology (Haimen, China). Western Bright ECL HRP substrate was purchased from Advansta (Menlo Park, CA, USA). 2-Methoxyestradiol (2-MeOE2) (CAS No. 362-07-2) was purchased from Selleck (Shanghai, China). STF-31 (CAS No. 724741-75-7) was obtained from MedChemExpress (Shanghai, China). Advanced glycation end products (AGE-BSA, No. 2221-BSA) and BSA (No. 2221-10) were purchased from BioVision (Milpitas, CA, USA). Low molecular weight heparin (LMWH) was purchased from Sigma-Aldrich Co., Ltd. (St. Louis, MO, USA). Enhanced cell counting kit-8 (CCK-8) (C0042), 4',6-diamidino-2-phenylindole (DAPI) (No. C1002) and ERS agonist tunicamycin (Ti, No. SC0393) was purchased from Beyotime Biotechnology (Shanghai, China) and ERS inhibitor 4-phenylbutyric acid (4-PBA) were purchased from Meilunbio Biotech (Dalian, China).

Collagenase II was obtained from Invitrogen (Carlsbad, CA, USA). DMEM (no glucose) medium (No. 11966025) was purchased from Gibco Co., Ltd (Grand Island, NY, USA). Fetal bovine serums (FBS) were supplied by Gibco (St. Louis, MO, USA). Polyclonal primary antibody information is as follows: anti-ACTB (No. AC026), anti-MMP13 (No. A11148), anti-ADAMTS5 (No. A2836), anti-78 kDa glucose-regulated protein (GRP78) (No. A0241), anti-activating transcription factor 6 (ATF6) (No. A0202), anti-TNF-α (No. A11534) and anti-IL-6 (No. A0286) were purchased from Abclonal Technology (Wuhan, China), anti-α1 chain of type II collagen gene (COL2A1) (No. 28459-1-AP), anti-aggrecan (ACAN) (No. 13880-1-AP), anti-GLUT1 (No. 21829-1-AP) were purchased from Proteintech Technology (Wuhan, China), anti-AGEs (No. bs-1158R) was acquired from Bioss (Beijing, China), anti-hypoxia-inducible factor (HIF)-1α (No. SAB2702132) and anti-NF-Kappa-B p65 subunit (NF-κB p65) (No. SAB5700780) were obtained from Sigma-Aldrich Co., Ltd. (St Louis, MO, USA). Secondary antibody information is as follows: Cy3 conjugated goat anti-rabbit IgG (H + L) (No. AS-1111) was obtained from Amejet Scientific (Wuhan, China); goat anti-rabbit and horseradish peroxidase-conjugated IgG (No. 4412) was obtained from Cell Signaling Technology (Danvers, MA, USA). Cy3 goat anti-mouse IgG (H + L) (No. AS008) and goat anti-mouse and horseradish peroxidase-conjugated IgG (No.AS064) were obtained from ABclonal (Wuhan, China). The other chemicals and agents were analytical grade.

2 Supplementary tables

Supplementary Table S1. The patients' information.

Patient ID	Age (y)	Gender	Complications	FBG [a] (mmol/L)	PBG [b] (mmol/L)	Height (cm)	Weight (kg)	BMI [c]
			Control group					
OA 01	65	Female	Hypoproteinemia, chronic sinusitis, tinea pedis	5.13	/	157	70	28.40
OA 02	67	Male	Foot moss, hypertension	6.35	/	175	90	29.39
OA 03	55	Female	Osteoporosis, hypoproteinemia	6.6	/	153	62	26.49
OA 04	52	Female	/	4.68	/	159	73	28.88
OA 05	62	Female	Upper respiratory tract infection, hypertension	4.61	/	/	/	/
OA 06	54	Female	/	5.29	/	/	/	/
OA 07	55	Male	Hypoproteinemia	6.55	/	/	/	/
OA 08	62	Female	Hypoproteinemia, moderate anemia	5.15	/	152	56	24.24
OA 09	68	Male	Senile valvular heart disease, senile osteoporosis	6.08	/	170	80	27.68
OA 10	70	Female	Chronic bronchitis, anemia, hypoproteinemia	4.94	/	155	61	25.39
			Diabetes group					
DM-OA 01	50	Male	Diabetes,	10.94	21.8	163	90	33.87

			hypoproteinemia, hepatic insufficiency, anemia					
DM-OA 02	69	Female	Type 2 diabetes, degenerative lumbar disc disease	10.3	8.9	165	53	19.47
DM-OA 03	63	Male	Type 2 diabetes	5.33	8.7	175	68	22.20
DM-OA 04	66	Female	Diabetes, hypertension	8.8	14.6	148	65	29.67
DM-OA 05	65	Female	Type 2 diabetes, hypertension	8.9	18.8	164	67	24.91
DM-OA 06	66	Female	Prediabetes, coronary atherosclerosis	6.56	10.3	159	72	28.48
DM-OA 07	62	Female	Type 2 diabetes	6.4	13.9	165	79	29.02
DM-OA 08	60	Female	Type 2 diabetes, hypertension	5.84	/	162	66	25.15
DM-OA 09	66	Female	Diabetes, dermatomyositis, interstitial pneumonia, hepatic cysts, herpes zoster sequelae, osteoporosis	6	7.8	160	80	31.25

DM-OA 10	63	Female	Diabetes, hypertension	9.8	11.9	150	76	33.78

[a] Fasting blood glucose.

[b] Postprandial blood glucose.

[c] Body mass index.

Supplementary Table S2. Comparison of basic parameters between two groups.

	Control group	Diabetes group	*P* value
Age(y)	61±6.55	61±5.23	0.4602
Gender (male, n(%)/female, n(%))	3(30)/7(70)	2(20)/8(80)	/
Height(cm)	160.14±8.88	161.10±7.72	0.8161
Weight(kg)	70.29±11.90	71.60±10.21	0.8103
BMI [a]	27.21±1.91	27.78±4.79	0.7707

[a] Body mass index.

3 Supplementary figures

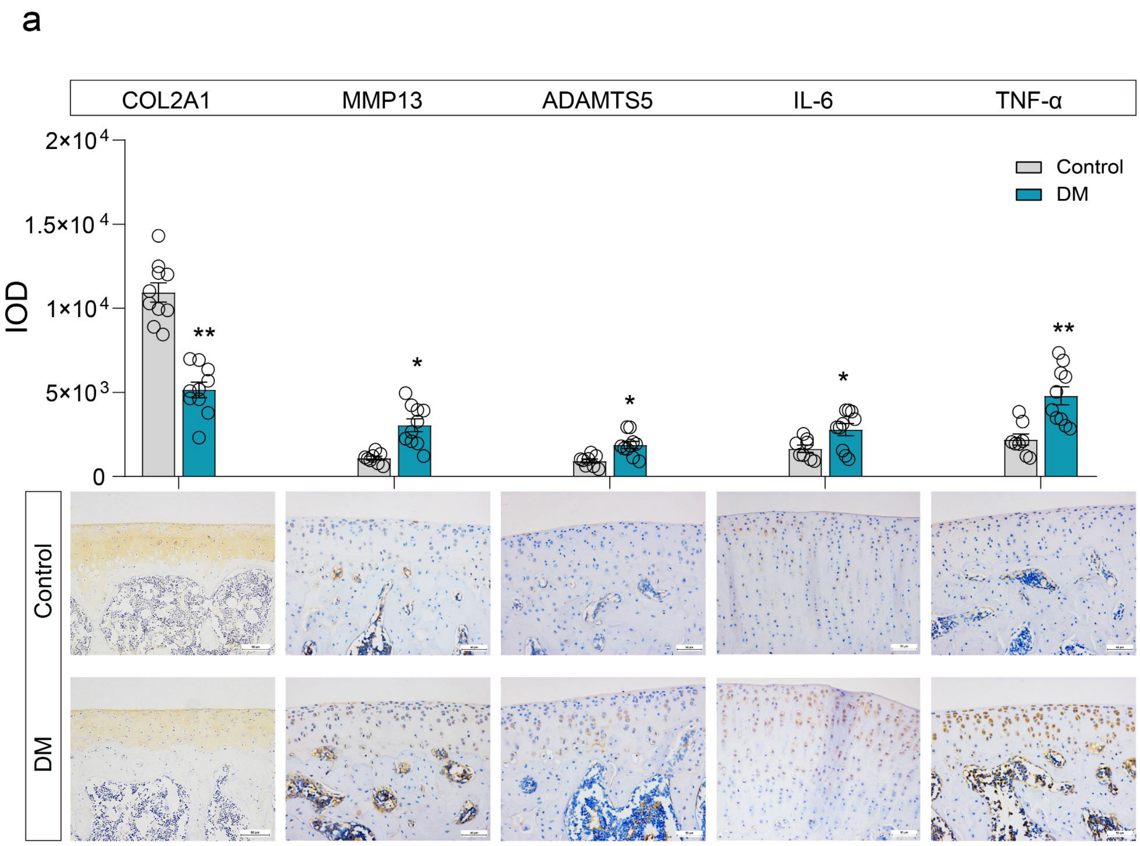

Supplementary Figure S1. The expression level of collagen, inflammatory and degradation factors in cartilage. a: Representative IHC images and the IOD value of COL2A1, MMP13, ADAMTS5, IL-6 and TNF-α of cartilage in the groups of control and DM, scale bar: 50 μm. Ten fields of view for each sample were selected for analysis. The values are the means±S.E.M., n=10. Mann-Whitney U test was used for statistical analysis. $^{*}P < 0.05$, $^{**}P < 0.01$ vs. Control.

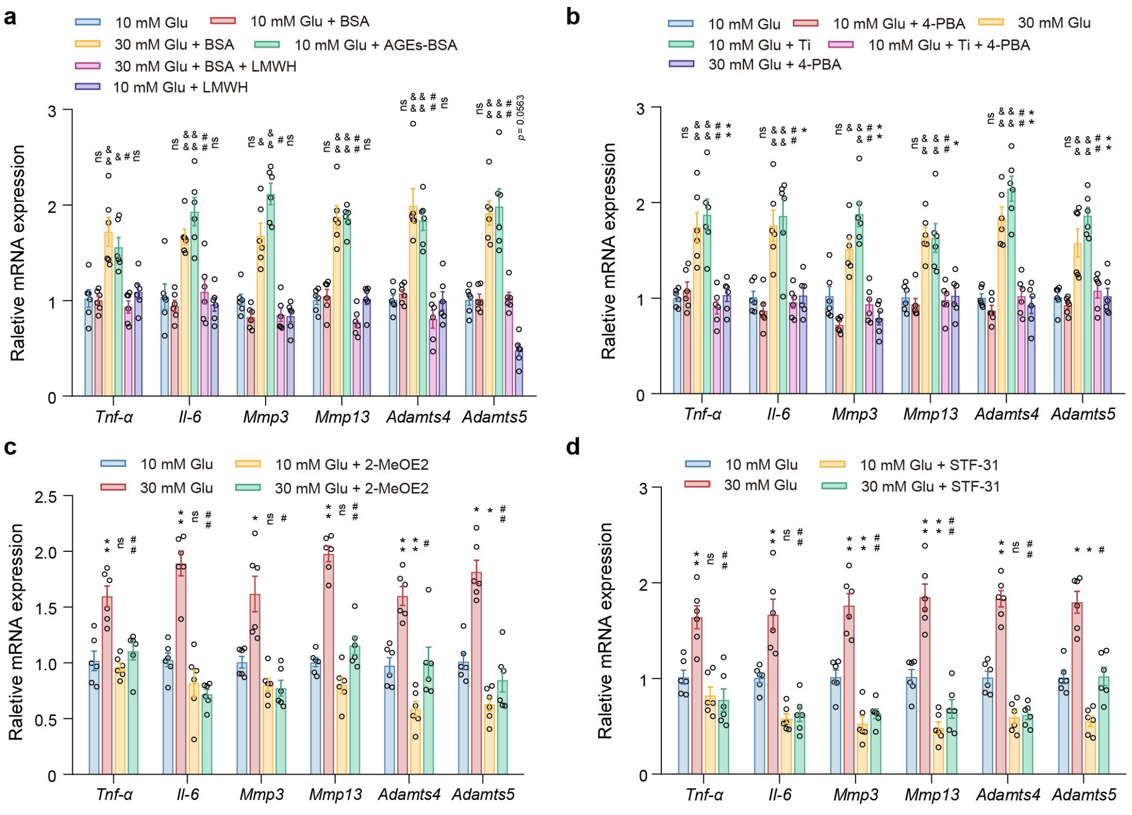

Supplementary Figure S2. The mRNA expression of inflammation factors of the FLSs. a-d: The mRNA expression of inflamation and degradation genes *Tnf-α*, *Il-6*, *Mmp3*, *Mmp13*, *Adamts4*, *Adamts5* of the FLSs, which was treated with 10mM glucose (Glu), 10mM Glu combined with 100 ng/ml BSA, 30mM Glu combined with 100 ng/ml BSA, 10mM Glu combined with 100 ng/ml AGE-BSA, 30mM Glu combined with 100 ng/ml BSA and 1 IU/ml LMWH, 10mM Glu combined with 1 IU/ml LMWH (**a**); 10mM Glu, 10mM Glu combined with 250μM 4-PBA, 30mM Glu, 10mM Glu combined with 1μg/ml Ti, 10mM Glu combined with 1μg/ml Ti and 250μM 4-PBA, 30mM Glu combined with 250μM 4-PBA (**b**); 10mM Glu, 30mM Glu. 10mM Glu combined with 2.5μM 2-MeOE2 and 30mM Glu combined with 2.5μM 2-MeOE2 (**c**); 10mM Glu, 30mM Glu, 10mM Glu combined with 0.5μM STF-31 and 30mM Glu combined with 0.5μM STF-31 (**d**). The values are the means ± S.E.M., n = 6. An unpaired Student' s *t* test was used for statistical analysis. $^{*}P < 0.05$, $^{**}P < 0.01$ vs. 10mM Glu group (**a, c, d**), or 30mM Glu group (**b**). $^{\&}P < 0.05$, $^{\&\&}P < 0.01$ vs.

10mM Glu combined with BSA group (**a**), or 10mM Glu group (**b**). $^{\#}P < 0.05$, $^{\#\#}P < 0.01$ vs. 30mM Glu group (**d**), or 30mM Glu combined with BSA group (**a**) or 10mM Glu combined with 4-PBA group (**b**).
